# Prediction of prognostic biomarkers for Interferon-based therapy to Hepatitis C Virus patients: a metaanalysis of the NS5A protein in subtypes 1a, 1b, and 3a

**DOI:** 10.1186/1743-422X-7-130

**Published:** 2010-06-15

**Authors:** Mahmoud M ElHefnawi, Suher Zada, Iman A El-Azab

**Affiliations:** 1 Informatics and Systems Department, Division of Engineering Research, National Research Centre, Tahrir Street, Cairo, Egypt; 2 Science and Technology Research Centre, American University in Cairo, Cairo, Egypt; 3 Biology Department, American University in Cairo, Cairo, Egypt; 4 Faculty of Computers & Information, Cairo University, Ahmed Zowail Street, Cairo, Egypt

## Abstract

**Background:**

Hepatitis C virus (HCV) is a worldwide health problem with no vaccine and the only approved therapy is Interferon-based plus Ribavarin. Response prediction to treatment has health and economic impacts, and is a multi-factorial problem including both host and viral factors (e.g: age, sex, ethnicity, pre-treatment viral load, and dynamics of the HCV non-structural protein NS5A quasispecies). We implement a novel approach for extracting features including informative markers from mutations in the non-structural 5A protein (NS5A), specifically its Interferon sensitivity determining region (ISDR) and V3 regions, and use a novel bioinformatics approach for pattern recognition on the NS5A protein and its motifs to find biomarkers for response prediction using class association rules and comparing the predictability of the different features.

**Results:**

A total of 58 sequences from sustained responders and 94 from non-responders were downloaded from the HCV LANL database. Site-specific signatures for response prediction from the NS5A protein were extracted from the alignments. Class association rules were generated (e.g.: sustained response is associated with position A2368T in subtype 1a (support 100% and confidence 52.19%); in subtype 1b, response is associated with E2356G/D/K (support 76.3% and confidence 67.3%).

**Conclusion:**

The V3 region was a more accurate biomarker than the ISDR region. Subtype-specific class association rules gave better support and confidence than profile hidden Markov models HMMs scores, genetic distances or number of variable sites, and would thus aid in the prediction of prognostic biomarkers and improve the accuracy of prognosis. Sites-specific class association rules in the V3 region of the NS5A protein have given the best support and confidence.

## Background

Hepatitis C virus (HCV) is a positive single stranded enveloped RNA virus belonging to the *Flaviviridae *family. It causes a persistent infection in immune-competent individuals [1]. Its major sequel is chronic active Hepatitis, liver fibrosis, cirrhosis, and hepatocellular carcinoma. It is a major concern for the future world health and development as it infects ~3% of the world population, and has no vaccine [2]. The only approved combined therapy of pegylated Interferon plus Ribavarin has limited success (80% for genotypes 2 & 3 and 50% in genotypes 1 & 4). Factors influencing response can be classified into viral, e.g. the baseline viral load, the genotype, and the viral quasispecies heterogeneity [3,4], and host which can be further divided into general parameters like age, sex, contamination period, liver fibrosis and cellular factors including genetic polymorphisms in cellular immunological proteins [5].

The NS5A is a multidomain phosphoprotein [6]; an integral part of the virus replicase complex[7]. It is involved in protein interactions with cellular proteins including cytokines, growth factors, oncoproteins, and signalling proteins, for a review see (Macdonald and Harris, 2004; and Reyes, 2002) [8,9]. NS5A also antagonizes numerous cellular pathways, including the antiviral interferon-α response pathway [10], and the jack stat pathway as part of the counter attack mechanisms employed by the virus [6]. Site-specific substitutions, higher genetic distances, and number of variable sites in the ISDR and the V3 regions as well as dynamics of the NS5A quasispecies after 4 weeks of therapy all showed correlation with favorable response to treatment [3,11,12]. This indicates the superiority of viral factors in determining the response result [13]. Genetic markers from the virus proteins are important to consider in view of the immunological nature of the Hepatitis C virus disease and the many reports confirming the importance of virus-immune system interactions for determining response outcome. But, first, some general comments on bioinformatics and data mining are necessary.

Data mining has been defined as the nontrivial extraction of implicit, previously unknown and potentially useful information from data. Classification is a classic data mining task, with roots in machine learning. Associative classification aims to detect relationships between categorical variables and large datasets. This enables identification of hidden patterns in large databases. Associative classification aims to discover a small set of rules in the database, called class association rules, to form an accurate classifier. The accuracy of the rules is measured by their support (relative frequency of the body or head of the rule) and confidence (conditional probability of the body given the head of the rule). Several algorithms have been implemented in association rule mining including the A-priori algorithm, the frequent item set mining algorithm (COFI) [14].

Bioinformatics as a subdescipline of data mining aims to improve our current knowledge and understanding of biological and molecular entities. Pattern recognition and representation of motifs is a fundamental problem in bioinformatics and bioinformatics for diseases. The need arises for methods that can find discriminative patterns between closely related set of sequences that exhibit different phenotypes such as virulence, drug resistance, etc. It is important to capture very subtle variations, which are discriminatively powerful, and leave out unimportant statistically insignificant variations between the sets of sequences. Different approaches for pattern representations from sequence data include regular expressions, position weighted matrices, sequence logos, profile hidden Markov models, etc. All these have been used in several motif databases (e.g.: PFAM [15]).

In silico approaches for motif identifications and representations have tremendously helped to guide in vitro and in vivo experiments. DNA and protein motifs that were discovered in silico could be verified as signatures for diagnosis, prognosis, and response to treatment for several pathogens and cancer.

In this work, we apply a novel bioinformatics approach for signature extraction, feature selection and classification; mining NS5A sequences from the HCV LANL database for response biomarker prediction. Informative class association rules with a certain threshold of support and confidence were generated to improve prognosis prediction. Pattern and variability analysis on the NS5A protein, and specifically on its most important motifs for IFN-therapy response, namely the ISDR and V3 regions are performed. The rational was that new molecular markers are needed to improve current criteria for IFN-therapy inclusion and prognostic prediction. An efficient comparison of the ISDR and V3 regions, and the three studied subtypes (1a, 1b, and 3a) was also due. Finally, a comparison between the results of the applied techniques is conducted.

Prognosis prediction will help in personalising the treatment for HCV patients, reducing the side-effects and high costs associated with IFN treatment therapy choice in view of the number of specifically targeted antiviral treatment (STAT-C) inhibitors that will be available soon. Up to our knowledge, pattern analysis and classification modelling in the study of response to IFN based treatment for HCV has not been done before. Our workflow for finding markers for response to IFN is composed of sequence collection and sorting, multiple sequence alignments, informative site identification and feature selection by using relative Shanon entropy, comparative sequence logos, and viral epidemiology signature pattern analysis (VESPA) for positional enumeration of amino acids in each group followed by generation of class association rules followed by selection of the best set of rules.

## Materials and Methods

### Sequence Collection and Analysis

We downloaded all available annotated HCV NS5A sequences from subtypes 1a, 1b, and 3a from the HCV LANL database [16] (See Table 1). Factors affecting the response to therapy like sex, age, basal viral load are randomly distributed. The sequences were annotated with information about genotype, country, and outcome of IFN therapy [17]. Sequence manipulations were performed using JALVIEW [18], and BIOEDIT [19]. They were grouped according to response type and subtype and the ISDR and V3 regions extracted. These regions were studied due to their significant correlations for response to therapy. Multiple sequence alignmnets were performed using MUMMALS [20] and sequences were compared against their consensus.

**Table 1:** Summary of sequence analysis and mean genetic distance

*Region*	*Geno-type*	*Responder group*	*# of sequences*	*# of variable sites*	*Mean Genetic Distance within group*	*Mean genetic distance between groups*
**NS5A**	1a	R NR	21 42	21 31	0.017 0.02	0.03
**NS5A**	1b	R NR	20 42	34 25	0.017 0.02	0.029
**NS5A**	3a	R NR	17 10	24 21	0.045 0.036	0.041
**ISDR**	1a	R NR	21 42	3 3	0.04 0.032	0.4849
**ISDR**	1b	R NR	20 39	12 13	0.054 0.052	0.054
**ISDR**	3a	R NR	17 10	10 1	0.04 0.018	0.028
**V3**	1a	R NR	21 42	6 6	0.249 0.193	0.216
**V3**	1b	R NR	20 42	19 17	0.272 0.166	0.227
V3	3a	R NR	10 17	6 13	0.062 0.065	0.051

The number of sequences in each genotype and response group is shown; variable sites and mean genetic distance are calculated. The number of variable sites was extracted from the alignments, and mean genetic distances within and between groups were calculated using the MEGA program.

### Variability and Phylogeny Analysis

Tree reconstruction for each subtype and region was done using the PROTDIST from the PHYLIP package [21], and the MEGA 4.0 software [22]. Genetic distances within and between groups were also calculated using the MEGA 4.0 program.

### Pattern Discovery and Feature Selection

Detecting the most statistically significant differences between the responder and non- responder groups was done using the VESPA [23] available from the HCV database which gave the most variable positions and their frequencies between responders and non-responders. Class association rules were generated from these tables. Relative Shanon entropy was calculated using the tool from the great facilities available from the HCV LANL database. Statistically significant variations were calculated with a threshold of P = 0.05.

The two Sample sequence logo [24] server was also used to identify and confirm significant variations between the two groups for each subtype and statistical significance assessed.

Profile HMMs for the responder and the non-responder groups were performed using the HMMBUILD program from the HMMER package [25,26]. Class association rules were generated for the sites with statistically significant variations between the two groups in both the comparative sequence logo and the relative Shanon entropy and those whose support and confidence are above 50% were retained.

The association rules were tested on a 10% subset of the sequences. The HMM search tool available from the HMMER package was also used to score the test sequences against a profile HMM and the prediction accuracy noted. The threshold genetic distances scores, HMM scores, and number of variable sites used for rule generation were inferred and class association rules were generated.

## Results

### Patients' Sequences and Variability Analysis

**Figure 1:**
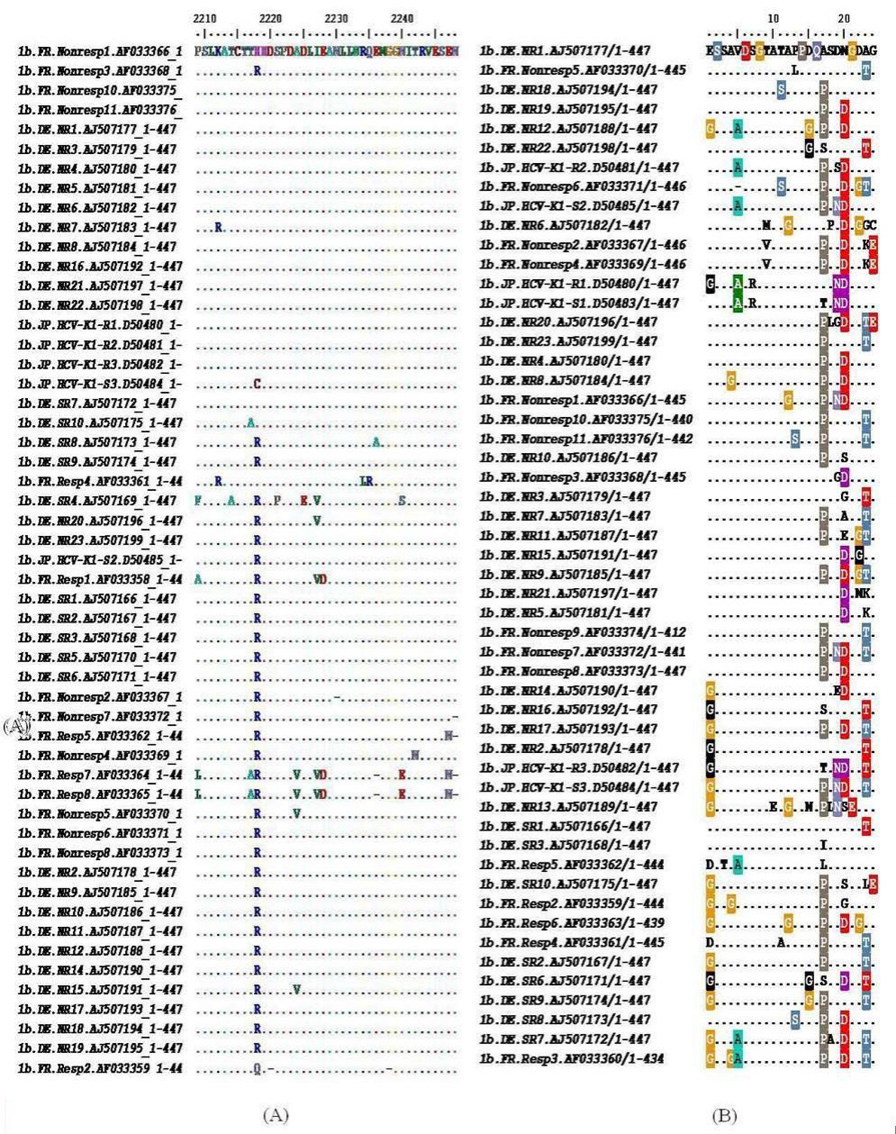
**Multiple sequence alignments of the ISDR and V3 regions for genotype 1b**. The responder strains are labelled with resp/sr, and non-responders with nonresp/nr. Dots represent conserved positions. 1A: ISDR amino acid sequences. 1B: V3 amino acid sequences. Both sequences are from responders and non-responders of genotype 1b.

A total of 58 sequences from sustained responder patients (R) and 94 sequences from non-responder patients (NR) were downloaded (Table 1). Protein multiple sequence alignments (MSAs) for sequences ofrespondersand non-responders were performed together and then sequences of the ISDR and V3 regions were extracted. The resulting MSAs are the corner stone for subsequent analysis and for building the classifier. Figure (1) shows the ISDR and V3 region alignments and conserved positions for subtype 1b. The resulting MSAs are the corner stone for subsequent analysis and for building the classifier. Distance based trees for each genotype and region are shown in additional file 1- figureS1. The trees show no clear clustering based on response, and the longer branches are mingled within both groups as previously deduced in similar studies[27]. There were no statistically significant correlation between the number of variable sites, genetic distances between responders and non-responders. For example, for the V3 region, there were 19 number of variable sites inresponders compared to 17 in non-responders (P = 0.86). In subtype 1a, the mean genetic distances are 0.249 in responders compared to 0.193 in non-responders (P = 0.35); while in subtype 1b, the mean genetic distances are 0.272 in responders compared to 0.166 in non-responders (P = 0.09). The number of variable sites and genetic distances in the V3 region were always higher than in the ISDR region (Table 1). Also, there was no statistical significance in number of variable sites, or genetic distances in the ISDR region and in the NS5A protein as a whole.

### Patterns Discovery and Recognition

**Figure 2:**
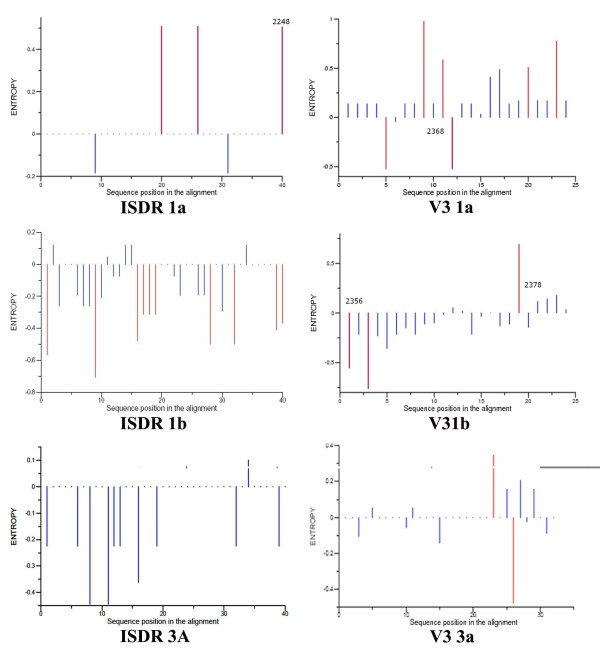
**Relative Shanon entropy between non-responders & responders in the ISDR & V3 regions of subtypes 1a, 1b and 3a**. It represents the difference between the positional entropy of the responders and non-responders (shown on the +ve and -ve scale respectively). It was calculated using the REL Entropy tool available from the great facilities at the HCV LANL database (significant positional variations between the two groups are labelled with red).

Positional variations in the ISDR and V3 regions were compared using a number of tools: VESPA, Relative Shanon entropy, and comparative sequence logos (see methods for elaboration). Signatures for response prediction were extracted from the MSAs using the VESPA tool (see additional file 1 -table S1and S2). Results reveal that in the ISDR region, the variations are small between the two groups of responders and non-responders. The relative entropy tool provided a different insight: The variations between the two groups are compared at every position, giving high scores for positions which are relatively variable in one group than the other. Results show that the variability in positions swings between the two response groups (sites with statistically significant variations (P < 0.05) are indicated with red in figure 2). Furthermore, the higher variability in the positions of the V3 region compared to the ISDR region can be deduced.

Comparative sequence logos confirm the results of VESPA and the relative Shanon entropy tool. The graphical motif representation enables a quick identification of positions that are clearly different by their length, and can therefore be incorporated in the classifier.

**Figure 3:**
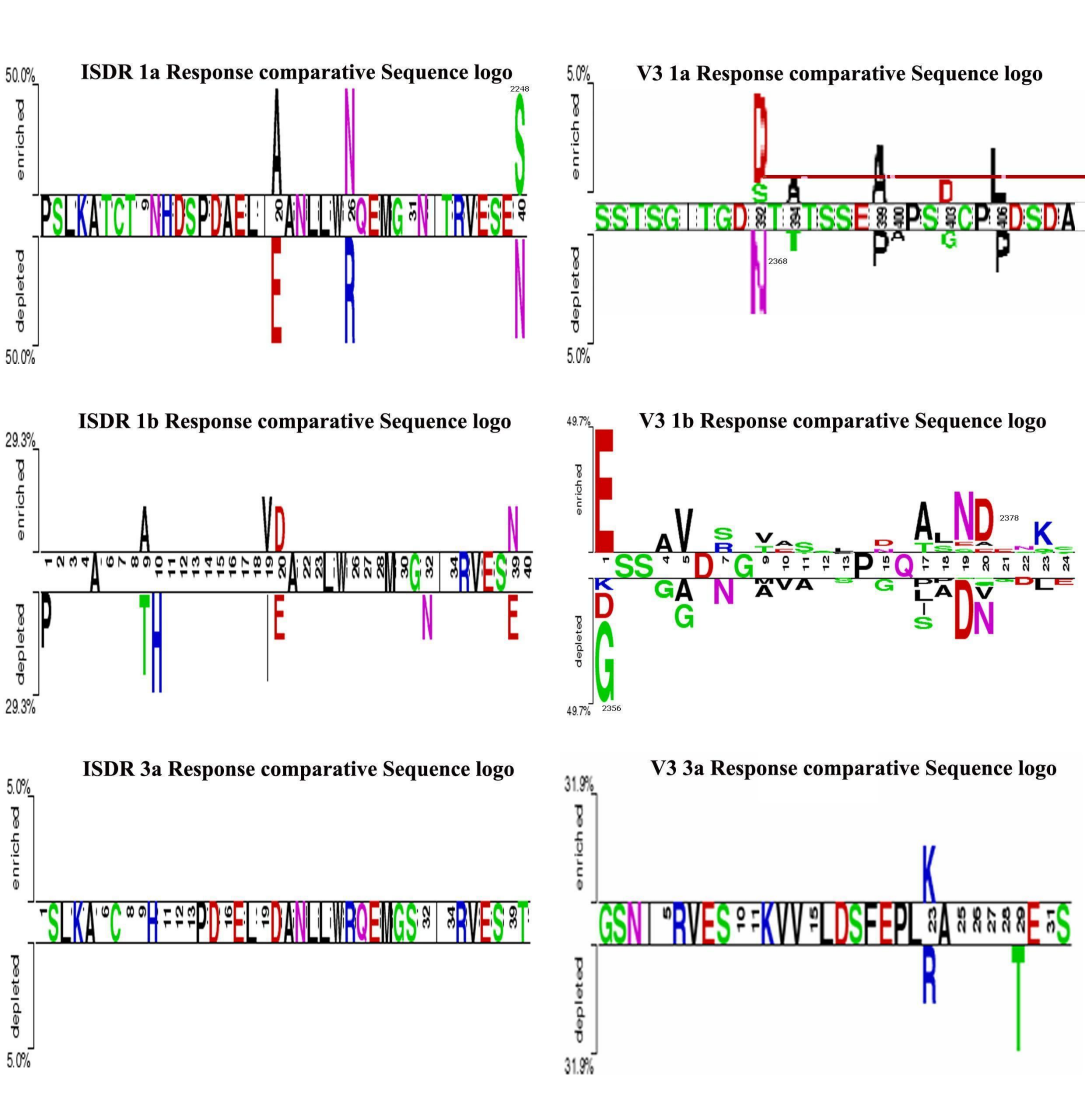
**Comparative sequence logos for the ISDR and V3 regions**. In the figure, the letters in the middle bar represent conserved positions. The totally empty positions represent variations within each group but no considerable variations between the two groups. The non-responders were set as the negative sample and the responders as the positive sample.

For the ISDR region: Subtype 1b showed the largest number of variations, which all clustered in the responders group (10 positions are indicated in Figure 2). Four positions coincided with the sequence logo results and statistically significant (2217, 2227, 2228 & 2247) (Figure 3). Thus, these positions are confirmed. Position 2228 is statistically significant in both subtypes 1a & 1b. For subtype 1a, there were 4 variable positions, 3 of them confirmed by the sequence logo (2228, 2234 & 2248) (Figure 3). There were no significant sites for subtype 3a.

In the V3 region, the following can be noted about site considerable variations between the two groups of responders and non-responders: There were four statistically significant sites (2356, 2358, 2374 and 2378) in the V3 region of subtype 1b which were confirmed by filtering results of both the relative Shanon entropy (Figure 2) and the comparative sequence logo (Figure 3). Similar analysis showed that there was no confirmed marker in subtype 3a and there were 4 positions in subtype 1a (2365, 2367, 2376,2379). Position 2378 was significantly variable between responders and non-responders in subtypes 1b and 3a.

There were 3 statistically significant variations in the IRRDR regions (2326, 2342 and 2349 in subtype 1a; 2332, 2348 and 2383 in subtype 3a).

For the whole of the NS5A protein, discriminative variations clustered in the IRRDR region and its flanking parts only.

No observable variations were present in other parts of the NS5A protein, and in the 2'5' OAS binding region.

Comparing genotypes 1 & 3, the number of variable sites, genetic distances, and statistically significant positions were lower in subtype 3a than 1a & b. The higher variability in subtype 1b could also be attributed to the diverse countries from which the patients came from.

### Evaluation and Comparison of Different Biomarkers

The class association rules for each subtype were generated from the VESPA, relative Shanon entropy, and comparative sequence logos results. The support and confidence of the class association rules have been calculated. The most informative rules with highest support and confidence are: In the V3 region, sustained response is associated with E2356G/D/K in subtype 1b (support 76.3% and confidence 67.3%), A2368T in subtype 1a (support 100% and confidence 52.19%). In subtype 1b, non-response is associated with wild type 2378T (support 50% and confidence 69%). In the ISDR region: In subtype 1a, non-response is associated with wild type 2248S (support 47.5% and confidence 95%).

We evaluated the genotype specific profile HMM models using responders and non-responders sequences. Similar scores for responder and non-responder sequences showed HMMs are not suitable for this kind of problem. The comparison of the different approaches for biomarker discovery is shown in Table 2. The table shows the higher accuracy of site-specific class association rules over other parameters.

**Table 2:** Summary comparison of the accuracy of different approaches used in the paper

*Method*	*Support*	*Confidence*
**Site-specific class Association rules**		
Wildtype 2378T in NR subtype 1b	50%	69%
A2368T in R in subtype 1a	100%	52.2%
E2356G/D in R in subtype 1b	76.3%	67.3%
		
**Number of variable sites** Three variable sites in R	100%	25%
Six variable sites in R	1.7%	100%
		
**Genetic distances** GD > 0.2 for the V3 region in R	55%	35%
		
Profile Hidden Markov Model **Score > 45 for R**	70%	20%

The different methods studied applied to the V3 region, which has been shown in the paper to be the most important for response prediction are compared in this table. The positive predictive value of the methods on the test set are shown.

## Discussion

Our objective was to extract patterns that can discriminate between two sets of phylogenetically close but functionally different sets of sequences. According to our results it is evident that variability is present in both groups; there were red lines and long letters in both response groups (Figures 2 and 3). Accordingly, an accurate measure which depends only on the variability would not be efficient in separating responders from non-responders. That's also why the profile HMMs, as maximum entropy models, didn't perform well.

The approach using class association rules extracted from the VESPA results (see additional file 1- table S1 and S2) and confirmed by relative Shanon entropy calculations and comparative sequence logos can help increase the sensitivity and specificity of genetic biomarker discovery in general. These class association rules, which are position and amino acid specific, proved more appropriate and gave high support and confidence. The associative classification technique was chosen because it builds more accurate and easily interpretable set of rules than traditional classification approaches [28,29]. Analysis of the genetic distance variations, VESPA, and relative Shanon entropy (Table 1, Figure 2 and Additional files 2 and 3) indicates the discriminative superiority of the V3 region over the ISDR region as a biomarker in the response to therapy problem. This was also confirmed by recent studies [30]. Subtype 3a showed lower overall variability and more homogeneity in both regions, with no statistically significant variations, thus indicating its higher rate for response. We correlated specific residues in the V3 region whose support and confidence exceeded both 50%. The previous structural and functional analysis [6] showed that the V3 region is 100% exposed, and contains a hot loop region, therefore highly ranking it as a protein binding motif. These mutations could limit the efficacy of the NS5A protein-host immune system proteins interactions in its counter attack mechanisms. Also, non-response was associated with specific amino acids in the V3 region which could be potential binding sites with the immune system proteins. Analysis of variability failed to accurately distinguish the response groups as these disordered proteins are inherently variable, with little effect by amino acid substitutions [31]. All three methods, VESPA, Shanon entropy, and comparative sequence logos, coincided in their results for the most important statistically significant variable positions between the two sets. An automated pipeline of analysis that incorporates these methods for signature extraction would aid in rapid sequence biomarker discovery in general. This can help physicians in drug type assessment as has been done with HIV drug resistance [32].

## Conclusions

We conclude that the IRRDR region is a better biomarker for therapy response than the ISDR region. Indicative biomarkers were extracted from subtypes 1a, 1b, and 3a, which showed significant variation between the two groups using a multi- bioinformatics approach for pattern analysis. Subtype 3a showed lower overall variability and more homogeneity in both regions, with no statistically significant variations, thus indicating its higher rate for response. Finally, comparing the results from pattern based approaches to analysis of variability, it is evident that rule generation methods, and pattern discovery are more reliable than noisy models (HMMs) and analysis of variability alone.

In conclusion, prognostic biomarkers have been extracted using this approach that would enhance prediction of response to IFN therapy in Chronic Hepatitis C patients.

## Competing interests

The authors declare that they have no competing interests.

## Authors' contributions

MMEH conceived of the study, participated in its design and coordination, performed the sequence analysis, discriminative pattern, classification and manuscript writing and revision. SZ helped in writing and revising the manuscript, and analysing the results. IAEA helped in the design, analysis, pattern recognition, writing and revision of the manuscript. All authors read and approved the final manuscript.

## Supplementary Material

Additional file 1 **Figure S1, Table S1, and Table S2**. **Figure S1: Distance-based tree of the ISDR and V3 regions for subtypes 1a, 1b, and 3a. 2A: **V3 1b nj tree **2B**: ISDR 3a nj tree. **2C**: V3 3a nj tree. **2D: **NS5A 1a nj tree. The trees were generated with the MEGA 4.0 program. The responder strains are labelled with resp/sr, and non-responders with nonresp/nr. Table S1: Substitutions frequencies for the ISDR region in the three subtypes using VESPA. The Tables were generated for each subtype separately using the VESPA tool from the HCV LANL database with the multiple sequence alignments of responders and non-responders as inputs, and significantvariations between the two groups were highlighted in the output. Table S2: Substitutions frequencies for the V3 region in the three subtypes using VESPA. The same procedure as above was repeated here for the V3 region. Click here for file
