# Supplementary material for: Prediction of prognostic biomarkers for Interferon-based therapy to Hepatitis C Virus patients: a metaanalysis of the NS5A protein in subtypes 1a, 1b, and 3a
Source: Virol J. 2010 Jun 15;7:130. doi: 10.1186/1743-422X-7-130 (PMC3238222; doi:10.1186/1743-422X-7-130)
Supplement: Additional file 1 — Figure S1, Table S1, and Table S2. Figure S1: Distance-based tree of the ISDR and V3 regions for subtypes 1a, 1b, and 3a. 2A: V3 1b nj tree 2B: ISDR 3a nj tree. 2C: V3 3a nj tree. 2D: NS5A 1a nj tree. The trees were generated with the MEGA 4.0 program. The responder strains are labelled with resp/sr, and non-responders with nonresp/nr. Table S1: Substitutions frequencies for the ISDR region in the three subtypes using VESPA. The Tables were generated for each subtype separately using the VESPA tool from the HCV LANL database with the multiple sequence alignments of responders and non-responders as inputs, and significantvariations between the two groups were highlighted in the output. Table S2: Substitutions frequencies for the V3 region in the three subtypes using VESPA. The same procedure as above was repeated here for the V3 region. [file 1743-422X-7-130-S1.PDF]

Additional files

Figure 4: **Distance-based tree of the ISDR and V3 regions for subtypes 1a, 1b, and 3a.**

2A: V3 1b nj tree **2B**: ISDR 3a nj tree. **2C**: V3 3a nj tree. **2D:** NS5A 1a nj tree.

The trees were generated with the MEGA 4.0 program. The responder strains are labelled with resp/sr, and non-responders with nonresp/nr.

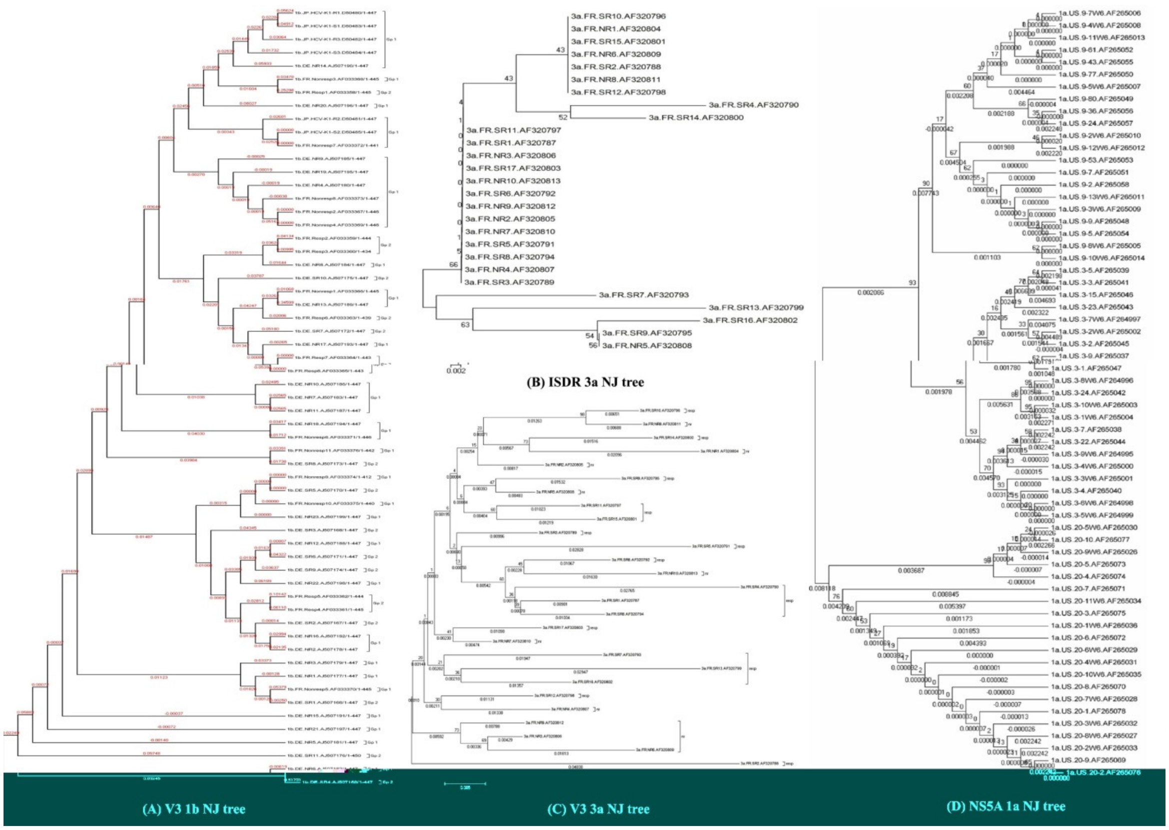

Table [3]: VESPA substitutions and frequency for the ISDR region

Position	Response	Genotype 1a ISDR region Frequencies using VESPA		Genotype 1b ISDR region Frequencies using VESPA		Genotype 3a ISDR region Frequencies using VESPA	
2209	Responder	P	0.976	P,L,A,F	0.810	P	1
2209	Non-Responder	P	0.512	P	0.974	P,V	0.941
2210	Responder	S	0.976	S	1	S	1
2210	Non-Responder	S	0.512	S,P	0.974	S	1
2211	Responder	L	0.976	L,S	0.905	L	1
2211	Non-Responder	L, A	0.512	L,S	0.974	L	1
2212	Responder	K	0.976	K,R	0.952	K	1
2212	Non-Responder	K	0.512	K,R	0.974	K	1
2213	Responder	A	0.976	A	1	A	1
2213	Non-Responder	A, U	0.512	A	1	A	1
2214	Responder	T	0.976	T,A	0.952	T	1
2214	Non-Responder	T,S	0.512	T	1	T,N	0.941
2215	Responder	C	0.976	C,Y	0.905	C	1
2215	Non-Responder	C	0.512	C,Y	0.974	C	1
2216	Responder	T	0.976	T,I	0.905	Q	1
2216	Non-Responder	T	0.512	T,I	0.974	Q,D,H	0.882
2217	Responder	A	0.976	T,A	0.762	T	1
2217	Non-Responder	A,V	0.488	T	1	T,S	0.941
2218	Responder	N	0.976	R,H,Q,T	0.619	H	1
2218	Non-Responder	N	0.512	R,H,C,Q	0.487	H	1
2219	Responder	H	0.976	H,Y	0.952	R	1
2219	Non-Responder	H, W	0.512	H,L,Y	0.949	R,E,G	0.882
2220	Responder	D	0.976	D	0.952	P	1
2220	Non-Responder	D,A,W	0.512	D,G	0.974	P,L	0.941
2221	Responder	S	0.976	S,P	0.952	H	1
2221	Non-Responder	S, F, A	0.512	S,C	0.974	H,Y	0.941
2222	Responder	P	0.976	P	1	P	1
2222	Non-Responder	P, F,A	0.512	P	0.974	P	1
2223	Responder	D	0.976	D	1	D	1
2223	Non-Responder	D, F, A	0.512	D,N	0.974	D	1
2224	Responder	A	0.976	A,D,V,F	0.762	A	1
2224	Non-Responder	A, F	0.512	A,V,F	0.923	A,V	0.882

2225	Responder	E	0.976	D,E	0.857	E	1
2225	Non-Responder	E	0.512	D	1	E	1
2226	Responder	L	0.976	L	0.905	L	1
2226	Non-Responder	L	0.512	L	1	L	1
2227	Responder	I	0.976	I,V	0.714	V	1
2227	Non-Responder	I	0.512	I,V	0.974	V,L	0.941
2228	Responder	E, A	0.5	E,D	0.857	D	1
2228	Non-Responder	E, A	0.488	E	1	D	1
2229	Responder	A	0.976	A	1	A	1
2229	Non-Responder	A	0.512	A	1	A	1
2230	Responder	N	0.976	N	0.952	N	1
2230	Non-Responder	N	0.512	N	0.974	N	1
2231	Responder	L	0.976	L	0.905	L	1
2231	Non-Responder	L	0.512	L	0.974	L	1
2232	Responder	L	0.976	L	1	L	1
2232	Non-Responder	L	0.512	L	1	L	1
2233	Responder	W	0.976	W	1	W	1
2233	Non-Responder	W	0.512	W	1	W	1
2234	Responder	R, N	0.5	R,L	0.905	R	1
2234	Non-Responder	R, N	0.488	R	1	R	1
2235	Responder	Q	0.976	Q,R	0.905	Q	1
2235	Non-Responder	Q	0.512	Q	1	Q	1
2236	Responder	E	0.976	E,A	0.857	E	1
2236	Non-Responder	E	0.512	E	1	E	1
2237	Responder	M	0.976	M	1	M	1
2237	Non-Responder	M	0.512	M	1	M	1
2238	Responder	G	0.976	G	0.857	G	1
2238	Non-Responder	G	0.512	G	0.947	G	1
2239	Responder	G	0.976	G	1	S	1
2239	Non-Responder	G,S	0.488	G	1	S	1
2240	Responder	N	0.976	N,E,S	0.857	N	1
2240	Non-Responder	N	0.512	N	1	N,D	0.941
2241	Responder	I	0.976	I	1	I	1
2241	Non-Responder	I	0.512	I	1	I	1
2242	Responder	T	0.976	T	1	T,V	0.9
2242	Non-Responder	T	0.512	T,N	0.974	T,V	0.941
2243	Responder	R	0.976	R	1	R	1
2243	Non-Responder	R	0.512	R	1	R	1
2244	Responder	V	0.976	V	1	V	1
2244	Non-Responder	V	0.512	V	1	V	1
2245	Responder	E	0.976	E	1	E	1

2245	Non-Responder	E	0.512	E	1	E	1
2246	Responder	S	0.976	S	1	S	1
2246	Non-Responder	S	0.512	S	1	S	1
2247	Responder	E	0.976	E	1	E	1
2247	Non-Responder	E	0.512	E	1	E,L	0.941
2248	Responder	N,S	0.524	N	0.810	T	1
2248	Non-Responder	N,S	0.488	N	0.974	T	1

*where there are more than one amino acid, the frequency value indicate the value of the first amino acid.

Table [4]: VESPA substitutions and frequency for the V3 region

Position	Response	Genotype 1a V3 region Frequencies using VESPA		Genotype 1b V3 region Frequencies using VESPA		Genotype 3a V3 region Frequencies using VESPA	
2356	Responder	S	1	G,D,E,K	0.556	G	1
2356	Non-Responder	S	1	E,G	0.775	G	1
2357	Responder	S	1	S,P	0.944	S	1
2357	Non-Responder	S	1	S	1	S	1
2358	Responder	T	1	S,A,P,T	0.778	N	1
2358	Non-Responder	T	1	S	1	N,D	0.978
2359	Responder	S	1	A,G	0.889	I	1
2359	Non-Responder	S	1	A,G	0.975	I	1
2360	Responder	G	1	V,A,G	0.667	T,V	0.963
2360	Non-Responder	G	1	V,A	0.850	T,V	0.978
2361	Responder	I	1	D,A	0.944	R	1
2361	Non-Responder	I, V	0.5	D	1	R	1
2362	Responder	T	1	S,N	0.889	V	1
2362	Non-Responder	T, M	0.952	S,R	0.950	V	1
2363	Responder	G	1	G,S	0.944	E	1
2363	Non-Responder	G	1	G	1	E	1
2364	Responder	D	1	T,A,M	0.889	S	1
2364	Non-Responder	D	1	T,V,M	0.925	S	1
2365	Responder	D,N,S	0.619	A,V	0.944	E,D	0.963
2365	Non-Responder	D	1	A,E	0.975	E,D,L	0.956
2366	Responder	T	1	T,A	0.944	T,S	0.963
2366	Non-Responder	T	1	T,S	0.950	T,S	0.978

2367	Responder	T	1	A,G	0.944	K	1
2367	Non-Responder	A, T	0.524	A,G	0.925	K	1
2368	Responder	T, A	0.810	P,S,L	0.944	V	1
2368	Non-Responder	T	1	P,S	0.925	V	1
2369	Responder	S	1	P,S	0.944	V	1
2369	Non-Responder	S	1	P	1	V	1
2370	Responder	S	1	D,G	0.889	I,V	0.963
2370	Non-Responder	S	1	D,G,N	0.925	I,V	0.911
2371	Responder	E	1	Q	1	L	1
2371	Non-Responder	E, D	0.976	Q	1	L	1
2372	Responder	P, A	0.571	P,A,S,I,L	0.667	D	1
2372	Non-Responder	P, L, A	0.905	P,A,S,T	0.625	D	1
2373	Responder	A, P, S	0.905	S,A,P	0.889	S	1
2373	Non-Responder	A	1	S,L,P	0.925	S	1
2374	Responder	P	1	D,G	0.944	F	1
2374	Non-Responder	P	1	D,N,G,E,S	0.7	F	1
2375	Responder	S	1	D,N,S,G,V	0.444	E	1
2375	Non-Responder	S	1	D,N,S,G,A,E	0.625	E	1
2376	Responder	G, D	0.81	G	1	P	1
2376	Non-Responder	G, D	0.976	G,E	0.975	P	1
2377	Responder	C	1	D,G	0.889	L	1
2377	Non-Responder	C	1	D,G,N	0.85	L	1
2378	Responder	P	1	A,T,L	0.5	R,K	0.778
2378	Non-Responder	P	1	T,A,K,G	0.45	R,K	0.956
2379	Responder	P, L	0.619	G,E	0.889	A	1
2379	Non-Responder	P	1	G,E,C	0.9	A	1

*where there are more than one amino acid, the frequency value indicate the value of the first amino acid.
